# Adult intussusception of the small intestine caused by cystic fibrosis: a case report, review of the literature, and guide for management

**DOI:** 10.1093/jscr/rjad574

**Published:** 2023-10-17

**Authors:** Yaw Adu, Brianna Wolkober, Esere Nesiama, Lori Thompson, Mujahed Laswi, Izi Obokhare

**Affiliations:** School of Medicine, Texas Tech University Health Sciences Center, 3601 4th St, Lubbock, TX 79430, United States; School of Medicine, Texas Tech University Health Sciences Center, 3601 4th St, Lubbock, TX 79430, United States; School of Medicine, Texas Tech University Health Sciences Center, 3601 4th St, Lubbock, TX 79430, United States; School of Medicine, Texas Tech University Health Sciences Center, 3601 4th St, Lubbock, TX 79430, United States; Department of Surgery, Texas Tech University Health Sciences Center, 1400 Coulter St S, Amarillo, TX 79106, United States; Department of Surgery, Texas Tech University Health Sciences Center, 1400 Coulter St S, Amarillo, TX 79106, United States

**Keywords:** intussusception, adult intussusception, cystic fibrosis

## Abstract

Intussusception, an uncommon but potentially severe condition primarily associated with infants and young children, can also present in adults, posing distinct challenges in diagnosis and treatment. This report presents the case of a 22-year-old male with cystic fibrosis, who developed intussusception due to severe constipation in his distal gastrointestinal tract. The patient’s initial presentation included abdominal pain, constipation, and abnormal laboratory results. Computed tomography scans revealed intussusception affecting the ascending colon and cecum, necessitating surgical intervention and subsequent bowel resection. In adults, the presence of intussusception often triggers suspicion of underlying pathological lead points. However, in this instance, the root cause was attributed to cystic fibrosis induced constipation. Current evidence suggests limited efficacy with conservative treatment, with bowel resection being the most definitive treatment option. Further research is warranted to establish comprehensive guidelines for managing this uncommon condition, particularly when intertwined with cystic fibrosis.

## Introduction

Intussusception, a rare but serious medical condition characterized by the telescoping of one segment of the intestine into an adjacent segment, typically affects infants and young children. However, it can occasionally manifest in adults, constituting 1–5% of bowel obstructions, and pose unique diagnostic and management challenges [1, 2]. While intussusception is uncommon in adults, it is typically associated with underlying pathologic conditions, which can be the intraluminal, mural, or extramural lead point for the intussusception [2]. We present the case of a 22-year-old male who presented with intussusception of the small intestine due to constipation of the distal gastrointestinal (GI) tract secondary to cystic fibrosis (CF).

## Case report

A 22-year-old male with a medical history of CF (F508/F508) presented with a 1-day history of persistent right lower quadrant (RLQ) abdominal pain. The patient complained of constipation despite taking lactulose. His symptoms worsened and he later developed obstipation. He denied any abdominal bloating, fever, chills, nausea or vomiting (n/v), and additionally, he denied previous abdominal surgical procedures.

**Figure 1:**
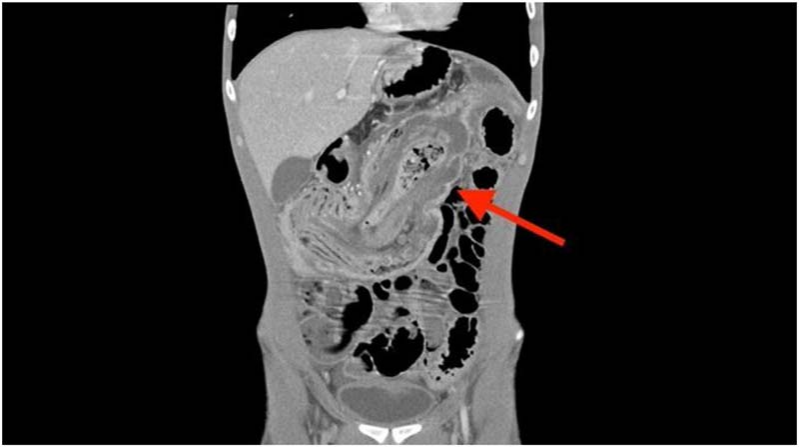
Coronal CT scan showing ileocolonic intussusception extending to the sigmoid colon.

**Figure 2:**
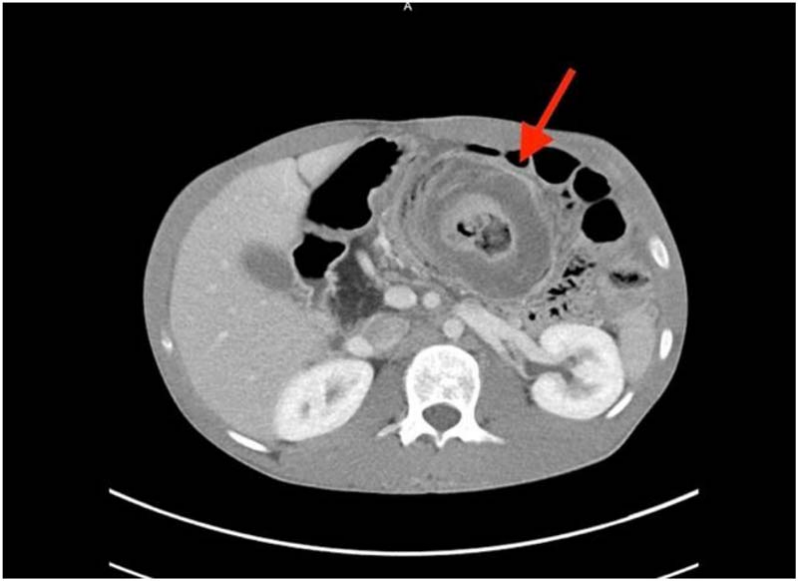
Axial CT scan showing characteristic target sign of bowel intussusception.

A coronal computed tomography (CT) scan with contrast showed a massive, small bowel to large bowel intussusception involving the majority of the ascending colon and portions of the cecum (Fig. 1). In addition, an axial CT scan showed the classic target sign associated with intussusception (Fig. 2). Due to the patient’s age, a pathologic lead point was suspected, hence surgery was consulted.

Exploratory laparotomy with lysis of adhesions, right hemicolectomy, and ileocolonic anastomosis was performed. During surgery, an ileocolic intussusception involving the cecum, appendix, and ascending colon into the mid-transverse colon was found. After multiple failed attempts to reduce the intussusception, the decision was made to proceed with right hemicolectomy. This portion of the bowel was resected, and once removed, a thick stool was found within the appendix, cecum, and ascending colon, raising suspicion that it served as the lead point for the intussusception. The excised right colon was sent for pathological examination. The pathology report revealed the presence of intussusception, along with mucosal inflammation, glandular changes, and serosal adhesions. Importantly, no signs of malignancy were detected.

Postoperatively, the patient was advanced to a regular diet, and by postoperative Day 5 he had no reports of n/v. He received a fleet enema and was discharged on postoperative Day 6 with MiraLax. At a 2-week follow-up appointment, the patient was tolerating his diet well with regular bowel movements.

Two months later, the patient presented to the emergency department following a 1-week history of alternating diarrhea and constipation and was discharged following treatment with MiraLax. During a follow-up appointment 1 week later, he reported experiencing intermittent episodes of alternating diarrhea and constipation, occasional loss of appetite, and episodes of abdominal cramping. He was advised to incorporate lactulose into his dietary regimen, along with a recommendation for a preventive colonoscopy to be scheduled within the next 6–12 months.

## Discussion

In the adult population, a majority of intussusception cases, ranging to as high as 90% in certain cases, are attributed to the presence of pathological lead points [1–3]. A large portion of these lesions, ~50 to 75%, typically manifest as benign neoplasms and predominantly originate within the small bowel [1–3]. In our patient’s case, the involvement of the small bowel was evident; however, the pathology reports revealed no malignancy, thereby necessitating the need for further investigation into an alternative causative factor.

It is worth noting that pediatric intussusception is frequently associated with predisposing factors such as CF [2]. Conversely, in the adult population, intussusception due to CF is exceedingly rare, with one study reporting that a mere 0.5% of all reported adult intussusception cases being attributed to CF [4]. Our patient’s medical history included CF with ongoing lung disease, chronic airway infections, pancreatic insufficiency, gastroesophageal reflux disease, and constipation. His background of CF prompted consideration of whether his intussusception could be attributed to CF-induced constipation resulting in the formation of a fecalith that subsequently telescoped into the small bowel.

The standard course of treatment for adult intussusception typically mandates bowel resection, especially given the high association rate between pathology and the presence of a lead point. Conservative treatment may be insufficient to manage patients, as evident from Table 1, where 8 out of 9 patients initially managed with conservative treatment experiencing symptom recurrence, with most eventually requiring surgical intervention [2]. A study conducted by Adewale *et al.* proposes that surgical intervention may not be the ideal approach for adult intussusception associated with CF. Instead, they recommend implementing a suitable regimen, such as pancreatic enzyme replacement therapy, to prevent constipation and the subsequent formation of a fecalith, which acts as a lead point. In the case of their patient, it was noted that this individual reported inconsistent adherence to replacement therapy, leading to recurrence of intussusception 7 weeks later [5]. Nevertheless, it is important to acknowledge that all reported cases treated conservatively except one experienced a recurrence of symptoms underscoring the potential necessity of bowel resection in all instances at some point.

**Table 1:** Case series of CF associated adult intussusception characteristics and outcomes.

Author	Age	Sex	CF-related comorbidities	Histologic findings	Conservative management	Surgical intervention	Outcome
Nash *et al.* [4]	37	F	Pancreatic insufficiency, CF-related diabetes, lung and heart disease requiring transplant	Tubulovillous adenomatous ileal polyps	Intravenous fluids, analgesia, antiemetics, laxatives, oral gastrografin, and phosphate enemas	Right hemicolectomy with primary anastomosis	Patient recovered well and was discharged
Nash *et al.* [4]	21	M	Pancreatic insufficiency, mild CF associated liver disease	Ilealtubular adenomas		Laparotomy and appendectomy	Eight months post op he was diagnosed with recurrent ileocolic intussusception and underwent right hemicolectomy
Nash *et al.* [4]	27	F	Pancreatic insufficiency, mildly impaired lung function	Hemorrhagic infarction		Ileocecectomy with ileo-ascending colon anastomosis	
Nash *et al.* [4]	28	M	Pancreatic insufficiency, lung disease requiring transplant	Marked bowel wall thickening with chronic eosinophilic inflammation	Managed conservatively with adequate symptom resolution and subsequent discharge		Two years later he was readmitted with CT scan showing an ileocolic intussusception with dilated loops of small bowel. A right hemicolectomy was performed.
^a^Nash *et al.* [4]	9	M				Laparotomy without bowel resection	
	19	M			Enemas and oral polyethylene glycol solution for DIOS		Continued to have intermittent colicky episodes of abdominal pain and vomiting over the next 2 years. CT scan showed mural thickening extension to the distal transverse colon
	24	M	Pancreatic insufficiency, chronic airway infection with *Burkholderia cenocepacia*	Edema and congestion		Right hemicolectomy	Good recovery
Nash *et al.* [4]	28	M	Pancreatic insufficiency, lung disease requiring transplant	Severely thickened colonic muscularis propria	Initially treated conservatively for DIOS with failure of symptom resolution	Right hemicolectomy	
Khera *et al.* [7]	18	M	Type 1 diabetes	Ischemic changes in the mucosa with eosinophilic material		Laparotomy with limited right hemicolectomy with primary anastomosis	Uncomplicated postoperative recovery
McIntosh *et al.* [8]	18	F	Moderately severe CF lung disease			Laparotomy	
Webb *et al.* [9]	18	M	Pancreatic insufficiency		Intravenous fluids and enemas		He was readmitted 1 month later with a laparotomy confirming an ileocolic intussusception that was later resected
Adewale *et al.* [5]	33	M			Polyethylene glycol, fluid replacement therapy, pancreatic enzyme replacement therapy, and bowel rest		At 7 weeks post discharge, the patient was readmitted with recurrent intussusception
Artul *et al.* [10]	26	M			Conservative treatment		Good clinical outcome
Gilchrist *et al.* [11]	35	F	Chronic *Pseudomonas aeruginosa* infection, CF-related diabetes	Polyps with moderately differentiated adenocarcinoma	Air insufflation	Hartmann’s procedure	Patient was without abdominal symptoms 13 months later
Thorsteinsson *et al.* [12]	36	F			Water-soluble contrast enema		Three weeks later, a colonoscopy showed no sign of malignancy or pathological lead points
Venkatasami *et al.* [13]	20	M		Focal neutrophilic infiltration of the surface epithelium with dilatation of crypts		Laparotomy-open appendicectomy	The intussusception had spontaneously resolved by the time of the procedure, but postoperatively, the patient’s abdominal symptoms improved
Cappell *et al.* [14]	19	F	Pulmonary bronchiectasis, chronic pancreatic insufficiency, and two admissions for RLQ pain due to distal intestinal obstruction syndrome (DIOS)	Infiltration of eosinophils		Laparoscopic appendectomy.	
Fishman *et al.* [15]	18	M		Thickened muscularis mucosae and fibrous expansion of the lamina propria with inflammation		Appendectomy	

^a^This corresponds to a single patient who had information for three different ages.

In cases of suspected intussusception, the initial diagnostic approach should involve an abdominal ultrasound examination. This method is known for its ability to reveal the characteristic bulls-eye or target appearance, although its accuracy is reduced in adults [2, 6]. Even if the ultrasound results are positive or inconclusive, a CT scan should follow, serving as the most accurate diagnostic test for adult intussusception. In adults, the CT scan typically also depicts the bulls-eye or target appearance, as seen in Fig. 2 [2].

Adult intussusception arising from CF induced constipation represents an exceedingly rare and diagnostically challenging entity. It is imperative to rule out other differentials of GI manifestations associated with CF, such as appendicitis, fibrosing colonopathy, and distal intestinal obstruction syndrome, through the appropriate use of imaging modalities [5]. Currently, this condition remains enigmatic, with limited studies available, rendering it challenging to provide comprehensive guidance on treatment. While adopting an adequate bowel regimen appears to be a promising approach for preventing recurrence in a conservative manner, bowel resection remains the sole definitive treatment option based on current knowledge and evidence.
